# Local myocardial stiffness variations identified by high frame rate shear wave echocardiography

**DOI:** 10.1186/s12947-020-00222-1

**Published:** 2020-09-29

**Authors:** Mihai Strachinaru, Johan G. Bosch, Arend F. L. Schinkel, Michelle Michels, Lida Feyz, Nico de Jong, Marcel L. Geleijnse, Hendrik J. Vos

**Affiliations:** 1 Erasmus MC Rotterdam, Cardiology, Postbus 2040, 3000 CA Rotterdam, The Netherlands; 2 Erasmus MC Rotterdam, Biomedical Engineering, Rotterdam, The Netherlands

**Keywords:** Naturally-occurring shear waves, Elastography, High frame rate tissue Doppler, Septal reduction

## Abstract

**Background:**

Shear waves are generated by the closure of the heart valves. Significant differences in shear wave velocity have been found recently between normal myocardium and disease models of *diffusely* increased muscle stiffness. In this study we correlate in vivo myocardial shear wave imaging (SWI) with presence of scarred tissue, as model for *local* increase of stiffness. Stiffness variation is hypothesized to appear as velocity variation.

**Methods:**

Ten healthy volunteers (group 1), 10 hypertrophic cardiomyopathy (HCM) patients without any cardiac intervention (group 2), and 10 HCM patients with prior septal reduction therapy (group 3) underwent high frame rate tissue Doppler echocardiography. The SW in the interventricular septum after aortic valve closure was mapped along two M-mode lines, in the inner and outer layer.

**Results:**

We compared SWI to 3D echocardiography and strain imaging. In groups 1 and 2, no change in velocity was detected. In group 3, 8/10 patients showed a variation in SW velocity. All three patients having transmural scar showed a simultaneous velocity variation in both layers. Out of six patients with endocardial scar, five showed variations in the inner layer.

**Conclusion:**

Local variations in stiffness, with myocardial remodeling post septal reduction therapy as model, can be detected by a local variation in the propagation velocity of naturally occurring shear waves.

## Introduction

Stiffness can be estimated in vivo by measuring the propagation velocity of externally induced shear waves travelling through tissue [1], the general principle being that shear waves travel faster in stiffer materials. Shear wave echography is an emerging diagnostic tool in radiology, capable of detecting relatively subtle changes in tissue elasticity. However, in cardiology, the dynamic stiffness of the myocardium over the heart cycle, as well as the limited acoustic access to the organ, present technical challenges to the implementation of cardiac elastography that are only addressed recently [2–7]. One technique relies on generating shear waves in the myocardium with an external source and detecting their propagation [8–10]. This approach is limited by the need for special equipment in order to induce and track these waves. Another technique, exploited in the current study, detects naturally occurring shear waves generated by the closing of the valves [3, 5, 11–14].

Studies have demonstrated that the global stiffness of the left ventricular wall can be determined by using shear wave imaging (SWI) [7, 12, 15].

Significant differences in shear wave velocity have been observed between normal myocardium and disease models of increased muscle stiffness, like amyloidosis [11], hypertrophic cardiomyopathy (HCM) [9, 10, 14, 16] and severe hypertension [17]. These studies focused on diffuse diseases of the myocardium, comparing shear wave velocity in pathological versus normal myocardium. The measured shear wave velocity has been referenced to invasive measures of left ventricular stiffness resulting from pressure/volume loops [8, 15], or morphological MRI studies [10], representing the global properties of the LV. However, in numerous disease states of the heart, the left ventricular myocardium is not homogenous, and the global properties of the cavity do not reflect regional tissue properties. Differences can be present between different myocardial segments, as would be the case for myocardial infarction scars, but also between different sub-regions within the same segments. Detecting these differences with echocardiography is currently feasible by using strain imaging [18–22], that is able to demonstrate segmental variations in tissue deformability, yet without being able to provide tissue stiffness characterization.

One of the main applications of SWI in other organs is generating tissue elasticity maps [1], capable of demonstrating local variations in tissue elasticity (elastograms) and giving diagnostic information beyond the normal B-mode imaging (ultrasound-based tissue characterization). High frame rate shear wave elastograms of skeletal muscles have been generated by using high-frequency linear probes at shallow depth [23]. In the heart however, additional challenges relate to depth, intercostal space opening and a continuously moving target. Currently no in vivo SWI data has been reported for a structurally inhomogeneous heart wall, where a local variation in tissue stiffness should result in a local variation in the propagation velocity of shear waves.

In this study, we aim to demonstrate that SWI can be used in the heart in order to give information on local tissue stiffness, by detecting local (subsegmental) variations in the velocity of naturally occurring shear waves.

## Methods

### Background

**Fig. 1:**
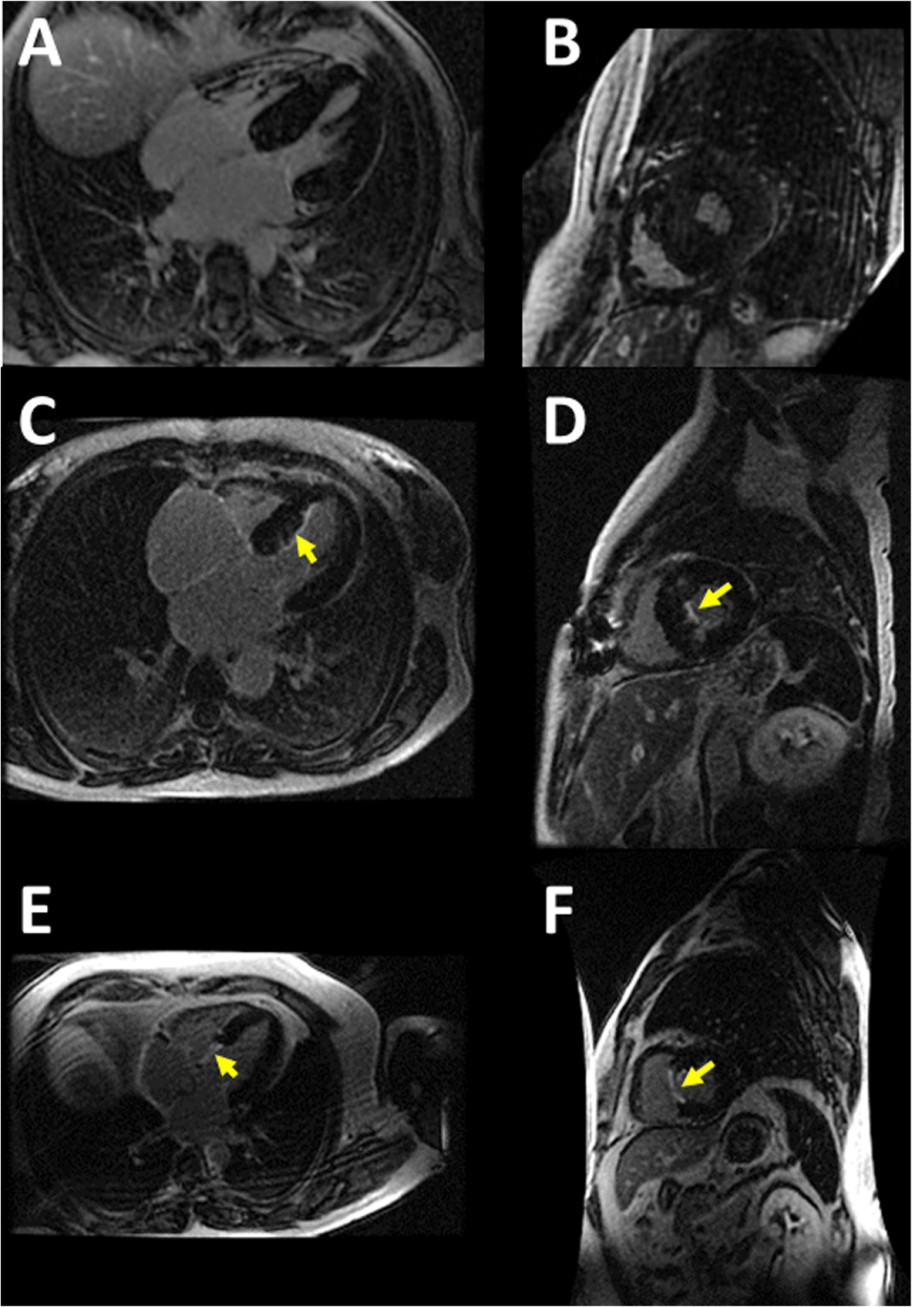
Delayed enhancement MRI images in our patients. **a**, **b**: 4-chamber view and corresponding short-axis view respectively, in a patient in group 2 (HCM patients without septal reduction therapy). No scar is detected; **c**, **d**: similar views in a patient in group 3 (HCM patients having undergone septal reduction therapy), demonstrating a thin subendocardial layer of fibrosis (arrow) resulting from surgical myectomy; **e**, **f**: same views in another patient in group 3, showing transmural delayed enhancement (arrow) in the basal septum, corresponding to transmural scarring resulting from interventional septal ablation

Ultrasound 2D, 3D and deformation imaging can detect localized intramyocardial variations and have been extensively validated in previous studies [21, 22, 24–26]. They were used as landmark references for SWI, for confirmation of the presence of local myocardial abnormalities in the investigated segments, making sure that scar tissue as visualized on MRI (Fig. 1) was indeed present in the 2D echocardiographic imaging plane used for SWI (parasternal long axis view) in each individual patient. This was done because surgical ablation scar in particular is expected to have a distribution that is independent of the myocardial vascularization (can be sub-segmental or trans-segmental).

Since shear waves in the myocardium could be affected by several factors such as shape, anisotropy, and inhomogeneity [27, 28] the precise quantitative relation between the naturally-occurring shear waves’ velocity and the intrinsic stiffness is difficult to establish directly. We therefore quantify only the propagation velocity of the shear waves, assuming a monotonic relation with the tissue stiffness [1, 29]. Actual stiffness quantification is outside of the scope of the current study. We aim to identify a localized variation in shear wave speed as proof for local variation in stiffness.

We have demonstrated before that HCM patients have significantly higher shear wave velocities as compared to normal individuals [14]. For the purpose of this study, we selected HCM patients having undergone septal reduction therapy. Fibrotic tissue resulting from septal reduction is stiffer than the adjacent myocardium and should induce a local increase in the propagation velocity of the shear waves. This will impact the full thickness of the septum for patients having a transmural scar but only the inner layer for non-transmural scar (see Fig. 1 for example MRI studies) [30]. These two models can be obtained after surgical myectomy (supposed to cause only endocardial scar formation) or septal alcohol ablation (supposed to cause endocardial or transmural myocardial scar formation).

Early experimental in vivo data shows that M-mode maps obtained from color TDI in the parasternal window can demonstrate transmurality of a myocardial scar and detect inter-layer differences, by looking at systolic deformation indices [31]. That method is dependent on local contractility (the distinction fails during active ischemia) and gives information over one thin short-axis slice corresponding to the axial M-mode line. SWI on the other hand, would potentially provide information on stiffness variation over the length of the basal septum, independent from its contractility. Conceptually however, it is not clear what to expect for the color TDI recordings of shear waves in a relatively thin visco-elastic medium having two adjacent layers of different properties. Therefore, we designed a two-step pilot study, starting with an in-vitro proof-of-concept and calibration experiment, and then applying it to live human subjects.

### In-vitro experiment

A slab-shaped dual-layer tissue phantom was created from 10% polyvinyl alcohol powder, 1% silicon carbide powder (50% SiC K-800, MTN-Giethoorn, the Netherlands; 50% SiC K-400, Cats, Hoogvliet, the Netherlands), 20% ethylene glycol (density 1.11 g/mol, Boom BV, Meppel, the Netherlands) and 69% distilled water. The stiffer layer (two cycles of freeze-thawing) had a final thickness of 8 mm, and the softer layer (one cycle of freeze-thawing)12 mm. This simulates an in vivo stiffer layer with a thickness less than 50% of the total wall.

We investigated this phantom using two different ultrasound scanners:

1. Philips iE33 system (Philips Medical, Best, The Netherlands) with S5–1 cardiac transducer, in high frame rate clinical TDI mode, tuned up to frame rates over 500 Hz (510 Hz at an imaging depth of 6 cm, corresponding to the in vivo parasternal long axis).
2. Aixplorer Multiwave system (SuperSonic Imagine, Aix-en-Provence, France), with a linear array S15–4 probe, used to generate an elastogram of the phantom and confirm the differences in local stiffness between the two layers.

Scanner 1 captured the shear waves in the slab that were induced by a Verasonics Vantage system with extended burst option (Verasonics, Kirkland, WA, USA), with a linear array L7–4 probe (Philips, Bothell, WA, USA) through acoustic radiation force (ARF). We refer to our previous study for detailed description of this method and settings [13].

**Fig. 2:**
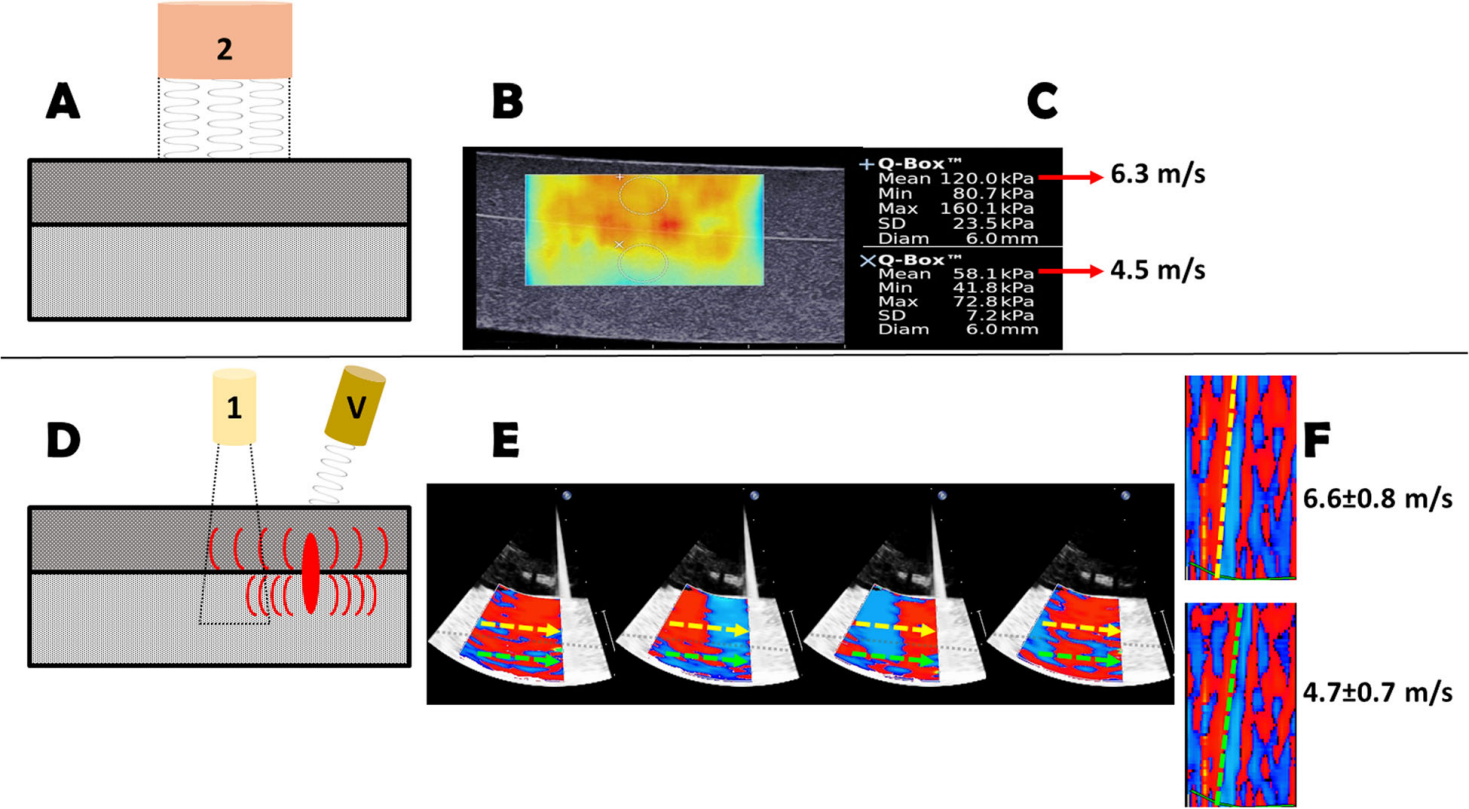
In vitro experiment: Dual-layer tissue phantom investigated using externally-induced shear waves. **a**: By using a clinical shear wave elastography system (scanner 2), color elastograms were generated (**b**); **c**: Regional stiffness quantification in the two layers, translated into shear wave velocity. The Q-boxes are manually placed in the middle of the layer-specific region; **d**: The same phantom imaged with a clinical high frame rate TDI system (scanner 1). Shear waves were induced by acoustic radiation force using a dedicated research system (V). **e**: a propagation difference is visible in the two layers. Because of the gain settings needed in the clinical system TDI, the interlayer separation is barely visible, and was marked with a dashed line; **f**: by computing the shear wave velocity along two M-mode lines, the values come very close to the expected shear wave velocities

The tissue phantom was placed in a water tank with the stiffer layer on top, as shown in Fig. 2. Using scanner 2 in shear wave elastography mode, we generated a color-coded elastogram of the two layers. Further, we used the Verasonics system to generate ARF pulses at a depth corresponding to the interface of the two layers, lateral to the field-of-view of scanner 1, which was used for detection, as previously described in bulk phantoms [13]. Two parallel M-mode lines were traced, one in the middle of each layer, both facing the excitation source.

### In vivo study

- *Group 1 (healthy controls): Healthy volunteers (N = 10)* aged 18 to 62 years. Subjects were excluded if one or more of the following criteria were present: a history of cardiovascular disease, systemic disease, the finding of cardiac abnormalities during the examination (including QRS duration over 100 ms), cardiovascular risk factors including hypertension (cutoff value 140/90 mmHg), diabetes mellitus or hypercholesterolemia, having breast implants or being pregnant. Professional athletes or morbidly obese (body mass index (BMI) > 40 kg/m^2^), were excluded.
- *Group 2 (HCM controls):* clinically stable *HCM patients (N = 10)* were recruited from the HCM outpatient clinic. Subjects were included if they had a definitive diagnosis of HCM [32] with basal septal involvement. Exclusion criteria were: prior septal reduction therapy, associated known coronary artery disease, more than mild valve disease. Systolic anterior motion of the mitral valve was not considered as exclusion criterion. These subjects were age-matched to group 3.
- *Group 3 (investigation group):* clinically stable *HCM patients (N = 10)* recruited from the HCM outpatient clinic, as defined above, but having undergone prior (more than 1 year before) septal reduction therapy, either by surgical myectomy (*n* = 8) or interventional septal ablation (*n* = 2). Each subject presented a known degree of myocardial scarring. If the MRI late gadolinium enhancement area covered the entire thickness of myocardium, segments were rated as having a transmural scar. Scarring less than 50% of the wall thickness was rated as subendocardial.

### Echocardiography

All echocardiographic studies were performed by one experienced sonographer (MS) using a Philips iE33 system (Philips Medical, Best, The Netherlands) with X5–1 cardiac transducer. Normal complete echocardiographic studies were performed, including 2D and 3D grey scale imaging. 2D imaging included the three standard apical views (four, two and three chamber) and parasternal long axis (PLAX). 3D volumes (four-beat full volume LV) were acquired from the apical window and analyzed using Qlab 9 postprocessing software (Philips Medical, Best, The Netherlands).

**The 3D volumes** were cropped using multislice views in the longitudinal and transversal plane, in order to detect myocardial scarring in the basal septum post septal reduction therapy. Scar was defined in grayscale 3D imaging as an area of localized myocardial thinning, with hyperechoic appearance, and the presence of localized wall motion abnormalities (Movie 1).

**Myocardial deformation** (current echocardiographic method detecting variations in local tissue properties) was analyzed by global and segmental longitudinal strain (Movie 2), by using a general independent post-processing platform (Tomtec Imaging System 4.6, Unterschleissheim, Germany). Global longitudinal strain (GLS) and longitudinal segmental strain (LSS) were computed in the apical view, according to current recommendations [33]. The results were represented in bull’s eye diagrams. The LSS in the basal and mid-wall septal segments were averaged and compared in the 4 and 3 chamber views (the views where septal segments are visible). For highest specificity, scar was defined as the presence of a myocardial segment with an area of localized myocardial thinning, with hyperechoic appearance, and the presence of localized wall motion abnormalities at 3D imaging plus LSS > − 5% (transmural scar) [21, 22, 24–26] or LSS > − 10%, but not > − 5% (possible scar without transmurality).

Data from 3D and deformation imaging were correlated and compared to the results from high frame rate echocardiography (see statistics section below for details).

In the **high frame rate echocardiography**, tissue velocities of the LV myocardium were sampled in Color Tissue Doppler (color TDI) in standard parasternal long axis view (PLAX) using the same Philips iE33 system, with an S5–1 transducer. As previously described [13, 14], TDI frame rates over 500 Hz were achieved by carefully tuning the imaging parameters (turning off all automatic smoothing and image enhancing applications, decreasing the 2D line density and adjusting the opening of the field of view, at the shallowest depth allowing to include the septal wall). These frame rates were sufficient to resolve the shear waves after aortic valve closure.

The TDI movies were stored in DICOM format for offline analysis. In order to discriminate shear waves from other events, the acquisitions were timed to the synchronous recording of both the electrocardiogram (ECG) and phonocardiography signal (PCG), the latter by using a Fukuda Denshi MA-300HDS(V) phonocardiography microphone.

**Fig. 3:**
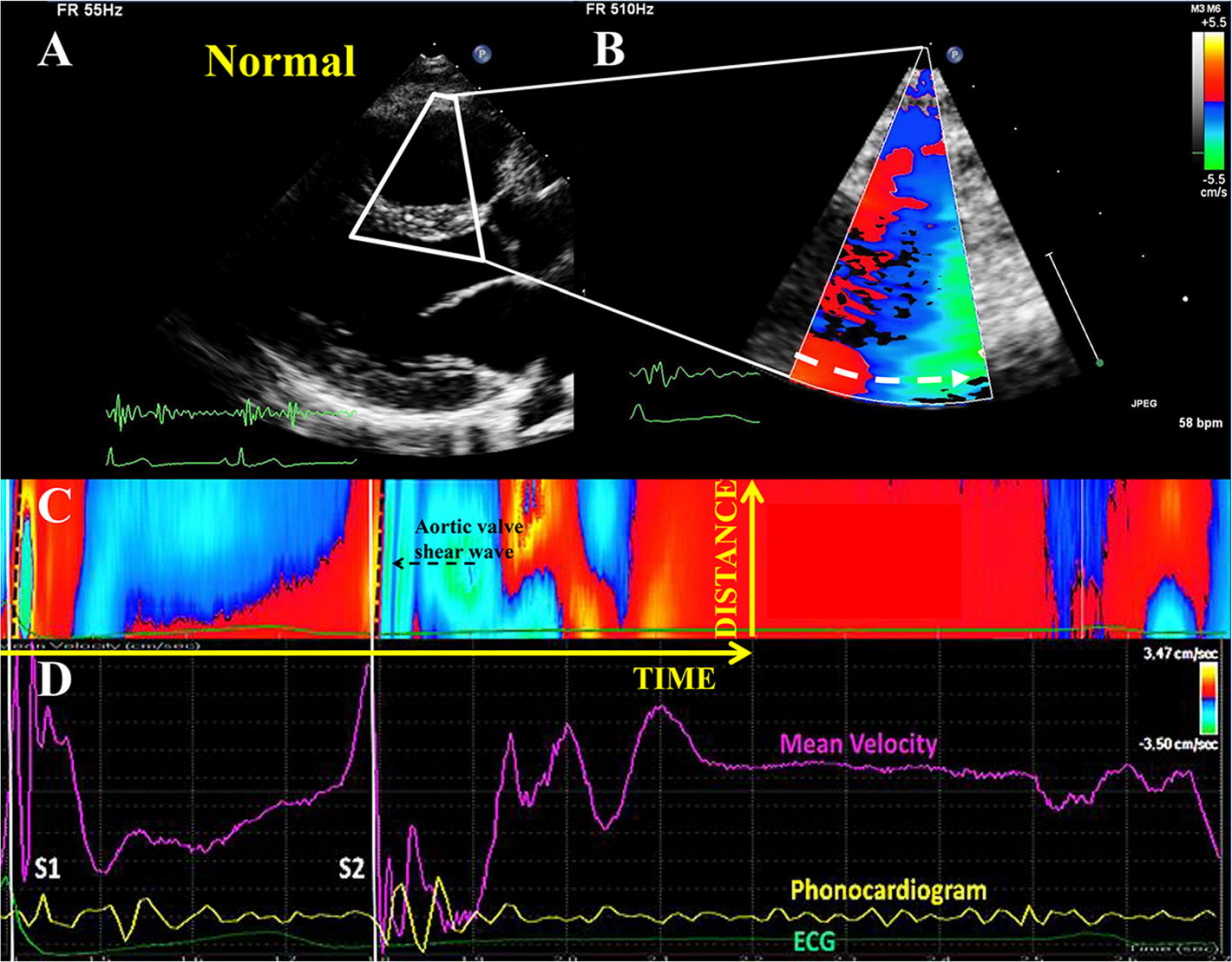
High frame rate color TDI shear wave tracking and analysis in the postprocessing software. **a**: classical parasternal long axis view and the focused TDI window over the interventricular septum. **b**: An M-Mode line is put in the septal wall, pointing towards the shear wave source. **c**: virtual M-mode map of a full heart cycle (reconstructed offline), at 510 Hz frame rate, demonstrating the shear waves after mitral and aortic valve closure (dotted slopes). The onset of the waves is marked with solid lines. The shear wave after aortic valve closure is pointed to by the dotted arrow; space and time directions are also indicated. **d**: mean tissue velocity curve as a function of time (averaged over the M-mode line, this velocity should not be mistaken with the shear wave propagation velocity), synchronous to the ECG (green) and PCG (yellow). The onset of both shear waves is synchronous to the onset of the respective heart sounds (S1, S2)

The DICOM TDI loops were processed using Qlab 9. A shear wave in the color TDI data is detected on the septal wall as a rapid up-and-down tissue displacement, visible in the form of a color shift from red to blue or blue to red, depending on the direction. Figure 3 demonstrates the recorded signals and our subsequent analysis. This pattern initiates at the exact visible moment of valve closure which also corresponds to the onset of the heart sounds in the PCG, and then propagates over the septal wall away from the valve towards the apex.

A curved virtual M-mode line can be traced along the LV wall (Fig. 3). For consistency, the arrow of the M-mode line always pointed towards the shear wave source, perpendicular to the wave front. The software provides a virtual M-Mode map, allowing to manually trace the leading slope of the propagating wave. As was previously described [13, 14, 16], this slope, in a time/distance M-mode map extracted from color TDI, represents the propagation velocity of the wave (Fig. 3). This velocity was computed mid-wall in our previous studies (Fig. 3). However, in the current study, we are interested in the existence of local variations of the propagation velocity of the wave, visible by changes in the slope of the wave front.

**Fig. 4:**
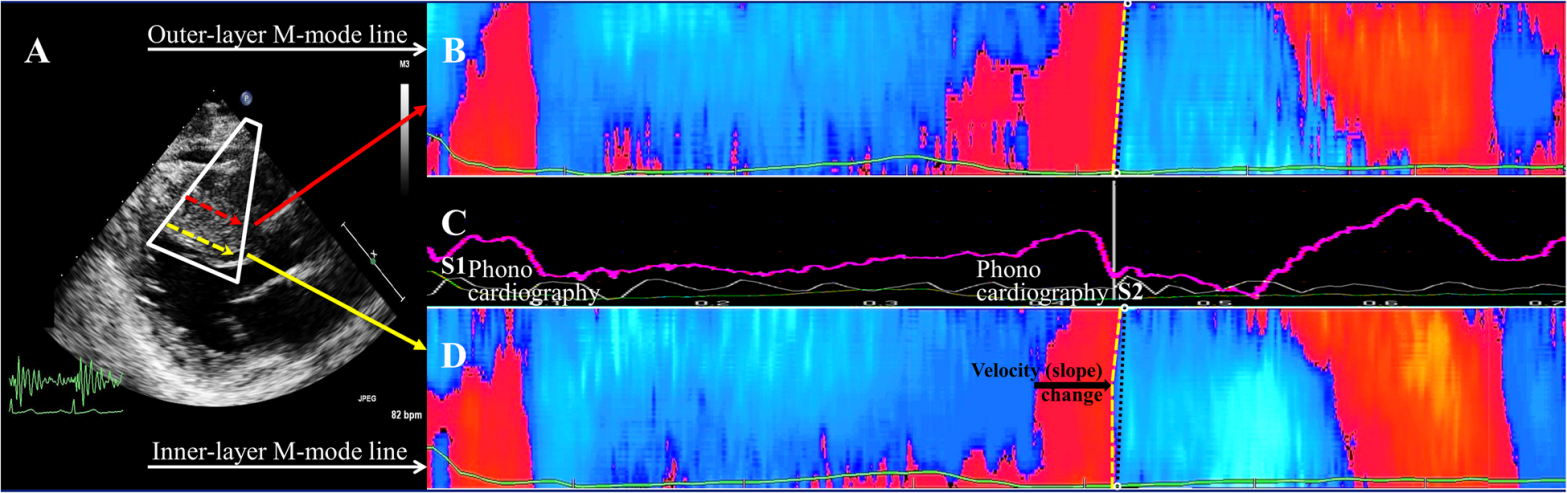
Explanation of the multi-layer TDI method used in this study, in one HCM patient having an inner layer scar. **a**: parasternal long axis and the focused TDI window over the interventricular septum; two M-Mode lines are traced, one in the outer layer and one in the inner layer, pointing towards the shear wave source. **c**: The mid panel is extracted from the Qlab postprocessing output and depicts synchronous ECG signal (green) and phonocardiogram (white). The average velocity line (pink line) is automatically generated by the software and should not be mistaken with the shear wave propagation velocity. Heart sounds are marked with S1 and S2. The onset of the second heart sound (S2) is marked with a white line and acts as reference for the onset of the aortic closure shear wave. **b**, **d**: local tissue velocity along the virtual M-mode lines traced in the outer layer (**b**) and inner layer (**d**). The entry and exit points of the shear wave along the two M-mode lines are marked with empty circles, connected by a white dashed straight line as reference. The aortic shear wave front slope is traced with yellow dashed lines. In panel B the wave front line is superposed on the reference line, but in panel D (inner layer) there is a visible shift in the slope, demonstrated by a deviation from the reference line

In this study the shear wave front propagating in the basal interventricular septum after the closure of the AoV was mapped along two virtual M-mode lines over a length of 3 cm, one in the inner (subendocardial) layer, the possible location of septal reduction scar, and one in the outer layer, as seen in Fig. 4a.

**Fig. 5:**
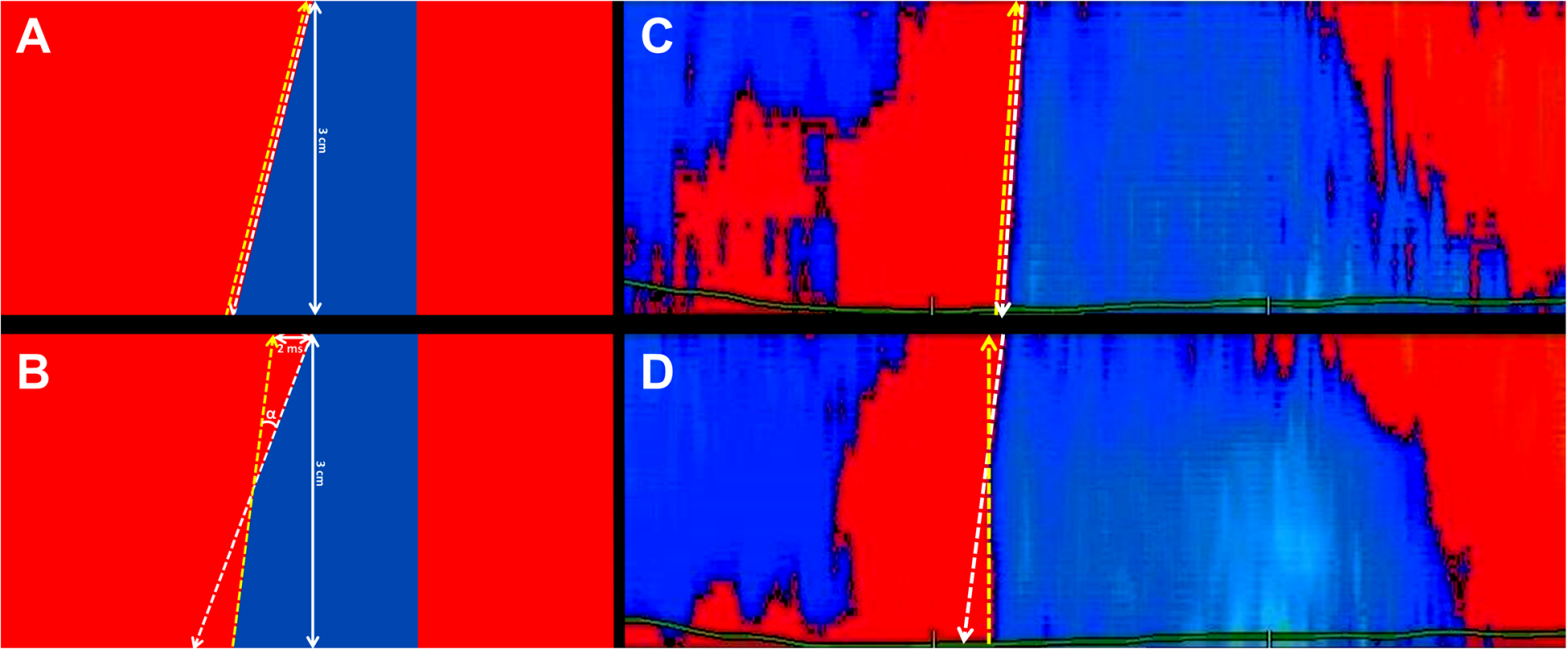
Shear wave velocity variation in a M-mode panel, theoretical model (**a**,**b**) and patient demonstration (**c**, **d**). **a:** uniform propagation of a wave (theoretical model). By tracing the wave front from the upper (white arrow) and respectively lower (yellow arrow) point we obtain two parallel lines; **b**: velocity variation along the 3 cm M-mode line (theoretical model). The upper point wave front (white arrow line) forms an angle (α) with the lower point arrow line (yellow line). This angle is resolved if the opening on either side is at least 2 ms (one time-frame at 500 Hz). It demonstrates a change in propagation velocity; **c**: outer layer tracing in a HCM patient. The wave front of the aortic shear wave is linear, the upper (white) and lower (yellow) point arrow lines are parallel; **d**: inner layer tracing in the same patient. There is a visible shift in slope, and the two lines intersect and form an angle. The spatial position of this intersection coincided with the transition scar-myocardium visible on 2D images

Manual tracking as allowed by the manufacturer-designed software is time consuming and prone to errors. Therefore, in order to reduce the variability in estimating the shear wave slope, we marked the entrance and exit points of the shear wave along the M-mode line (Fig. 4). We then qualitatively assessed the slope variation of the wave front in the basal septal segments by tracing two different straight lines in the M-mode maps, starting from the lower (entry) and respectively the upper (exit) points of the wave along the M-mode line, and following the shear wave front. If the upper and lower front lines were parallel, we scored this segment of tissue as having uniform velocity of the shear wave along the virtual M-Mode line (Fig. 5a). If the upper and lower slopes intersected, forming an angle with an opening of more than one time-frame (2 ms at 500 Hz) over the 3 cm line (Fig. 5b), the propagation velocity was considered to vary significantly within the detection capability of our TDI method along the length of the M-mode line. The angle threshold was derived by using the segment length (3 cm) and the time resolution of 2 ms at 500 Hz. For a wave travelling with around 5 m/s over 3 cm the detectable velocity variation is 1.5–2 m/s. The variability of our clinical TDI system was discussed and quantified in previous in vitro work [13]. In the presence of such a velocity variation we scored the underlying tissue as having a variation in its mechanical properties, i.e., scored as scar. This variation was scored as transmural if present in both layers.

### Statistical analysis

Continuous variables were represented as mean ± standard deviation (SD). Categorical data are presented as absolute number and percentages. For comparison of continuous variables we used the one-way ANOVA analysis. When significant differences were found, post-hoc analysis using the Tukey test was performed in order to assess differences between groups. Inter-observer variability for the qualitative assessment of the front of the shear waves was estimated on the HCM patients group, by using Kappa statistics [34] to compare between the readings from two different observers (one experienced cardiologist with knowledge of the method (MS) and one interventional cardiology fellow (LF) without prior knowledge of shear wave imaging).

All the statistical analyses were performed using the Statistical Package for Social Sciences version 21 (IBM SPSS Statistics for Windows, Armonk, New York, USA). Testing was done two-sided and considered significant if the *p* value was smaller than 0.05.

## Results

### In vitro study

By imaging the tissue phantom with scanner 2, we obtained elastograms of the two layers (Fig. 2). These color-coded elastograms clearly showed a difference in stiffness, irrespective of the position of the two different layers. The Young’s modulus measured in the stiffer layer was 120 kPa corresponding to a shear modulus of 40 kPa, thus a shear wave velocity of around 6.3 m/ s[1]. In the softer layer, Young’s modulus was 60 kPa, corresponding to a shear modulus of 20 kPa and a shear wave velocity of 4.5 m/s (slow layer).

In the next step, an ARF push was induced in the tissue phantom, and the resulting shear waves detected simultaneously in the two layers with scanner 1. We noticed a difference in the propagation of the shear wave between the two layers (Fig. 2). The average velocity computed with scanner 1 in the fast layer (*N* = 6 separate acquisitions) was 6.6 ± 0.8 m/s, and in the slow layer 4.7 ± 0.7 m/s.

### In vivo study

Baseline characteristics of the three study groups are presented in Table 1. Age, body mass index and septal thickness were significantly different in the HCM groups 2 and 3 as compared to the normal volunteers (group 1), but statistically similar between groups 2 and 3 (Table 1). Ejection fraction was significantly lower in group 3 (septal reduction group) as compared to both group 1 and 2.

**Table 1:** baseline characteristics of the three study groups

Category	***1 = Normal volunteers*** ***N = 10***	***2 = HCM patients without septal reduction*** ***N = 10***		***3 = HCM patients post septal reduction*** ***N = 10***		***ANOVA*** ***p*** ***2*** vs ***3***
			***ANOVA*** ***p*** ***1*** vs ***2***		***ANOVA*** ***p*** ***1*** vs ***3***	
**Age**	37 ± 14	51 ± 11	**0.002**	54 ± 10	**0.0001**	0.75
**Male gender**	50%	90%	0.09	60%	0.8	0.3
**BMI**	22 ± 2	28 ± 5	**0.002**	27 ± 5	**0.02**	0.7
**Systolic blood pressure [mmHg]**	118 ± 16	136 ± 12	0.06	130 ± 22	0.2	0.7
**Diastolic blood pressure [mmHg]**	70 ± 9	80 ± 12	0.4	76 ± 11	0.5	0.9
**Septal thickness [mm]**	8 ± 1	19 ± 5	**< 0.0001**	17 ± 4	**< 0.0001**	0.8
**Ejection fraction [%]**	67 ± 5	65 ± 9	0.9	53 ± 14	**0.01**	**0.03**

*BMI* Body Mass Index; *HCM* Hypertrophic cardiomyopathy

### 3D echocardiography

In the normal volunteers and HCM patients without septal reduction no scar was detected. In the group 3 HCM patients post septal reduction three patients (2 alcohol ablations and one surgical) had a transmural scar in the basal septum, six patients had only an endocardial scar and in 1 patient no scar was detected with 3D echocardiography (Table 2).

**Table 2:** Results from 3D echocardiography, deformation imaging and shear wave imaging in the 10 patients post septal reduction therapy (group 3)

	No scar detected	Endocardial scar	Transmural scar
**Longitudinal strain**	0	7	3
**3D echocardiography**	1	6	3
***Scar present in the PLAX imaging plane***	*1*	*6*	*3*
**Shear wave imaging (PLAX imaging plane)**	2	5^a^	3^b^

^a^All patients had abnormalities in the inner myocardial layer only

^b^All patients had abnormalities in both myocardial layers

*PLAX* Parasternal long-axis view of the left ventricle

### Deformation imaging

**Fig. 6:**
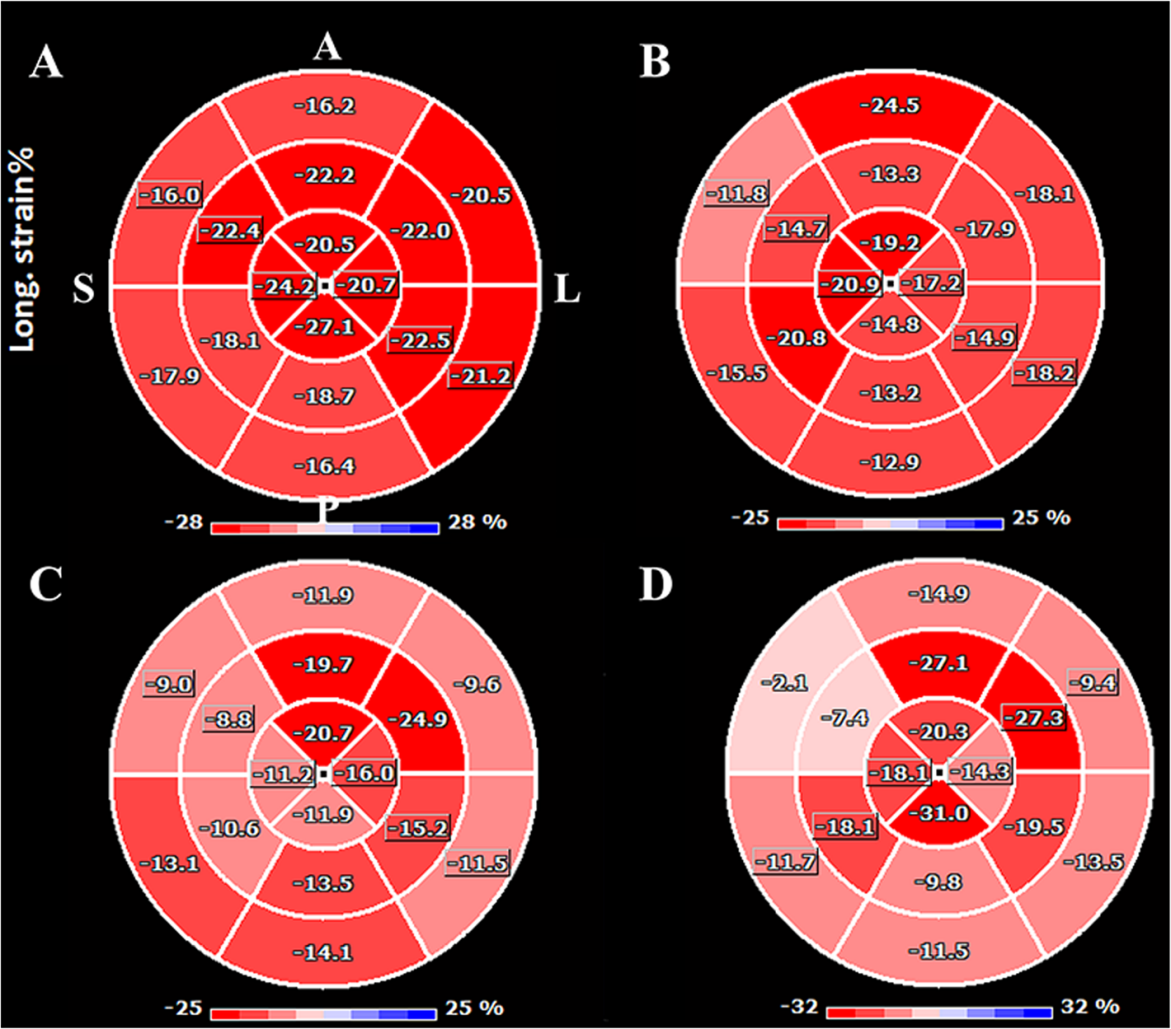
Strain results, represented as bull’s eye diagrams, by using a 16-segments model. **a**: normal volunteer. Local segmental strain (LSS) is homogeneous and normal in all the segments; **b**: HCM patient without septal reduction. LLS is globally lower than in the normal individual, with even lower values in the anteroseptal and inferior segments; **c**: HCM patient post septal reduction, having an inner layer scar visible on 2D and 3D echo. Anteroseptal segments have local strain values of > − 10%; **d**: HCM patient with transmural scar: anteroseptal segments have LSS values> − 5%

Global longitudinal strain (GLS) (Table 3) was significantly lower only in group 3, as compared to group 1 and group 2. GLS was not significantly lower in group 2 as compared to group 1. However, average septal LSS in 4 and 3 chamber views were significantly lower in the two HCM groups (Fig. 6, Table 3) when compared to normal volunteers (group 1). A significant difference existed between the average septal LSS between group 2 and 3 in apical 3 chamber view (− 16 ± 4% vs − 10 ± 4% respectively, *p* = 0.01), but not in 4 chamber view (− 16 ± 4% vs − 13 ± 4% respectively, *p* = 0.2).

**Table 3:** 2D longitudinal strain analysis in the study group

***Parameter***	***Group 1*** *Normal volunteers*	***Group 2*** *HCM patients without septal reduction*	*ANOVA* ***p*** *1* vs *2*	***Group 3*** *HCM patients post septal reduction*	*ANOVA* ***p*** *1* vs *3*	*ANOVA* ***p*** *2* vs *3*
**GLS (%)**	-20 ± 2	−19 ± 4	0.9	−14 ± 5	**0.001**	**0.004**
**Average septal**^a^ **LSS** **4 chambers (%)**	−23 ± 3	−16 ± 4	**< 0.0001**	−13 ± 4	**< 0.0001**	0.2
**Average septal**^a^ **LSS** **3 chambers (%)**	−20 ± 4	−16 ± 4	**0.04**	−10 ± 4	**< 0.0001**	**0.01**
**Any**^a^ **septal LSS > − 10%** **(number)**	0	0	–	10 subjects	–	–
**Any**^a^ **septal LSS > −5%** **(number)**	0	0	–	3 subjects	–	–

^a^: *the septal basal and mid-wall segments were considered, in the 4 chambers and 3 chambers views. GLS* Global Longitudinal Strain; *LSS* Longitudinal segmental strain

Furthermore, only in group 3 septal segments with LSS > − 10% were present in every subject (Table 2).

### Shear wave imaging

In high frame rate TDI, shear waves were visible in all 30 subjects after the closure of the AoV in the interventricular septum, synchronous to the onset of the second heart sound. In group 1 (normal) and group 2 (HCM without reduction) the slope of the wave front as traced from the upper and respectively lower point were parallel, no variations in shear wave velocity were detected.

The aortic valve closure shear wave, measured mid-wall as demonstrated in Fig. 3, had a velocity of 3.6 ± 0.4 m/s in healthy volunteers and 5.3 ± 1.0 m/s in HCM patients in group 2. In group 3, the mid-wall measurement was performed in non-ablated myocardial segments, and the velocity obtained was 5.3 ± 0.7 m/s, *p* = 0.9 versus group 2 (Table 4).

**Table 4:** Results of the shear wave imaging study

Category	***1 = Normal volunteers*** ***N = 10***	***2 = HCM patients without septal reduction*** ***N = 10***		***3 = HCM patients post septal reduction*** ***N = 10***		***ANOVA*** ***p*** ***2*** vs ***3***
			***ANOVA*** ***p*** ***1*** vs ***2***		***ANOVA*** ***p*** ***1*** vs ***3***	
**Color TDI frame rate [Hz]**	528 ± 22	530 ± 25	0.9	514 ± 17	0.4	0.3
**Aortic shear wave velocity in non-ablated segments [m/s]**	3.6 ± 0.4	5.3 ± 1	**< 0.0001**	5.3 ± 0.7	**< 0.0001**	0.9
**Slope/velocity shift present in any layer**	0	0		8		

*HCM* Hypertrophic cardiomyopathy; *TDI* Tissue Doppler Imaging

In group 3 (HCM post septal reduction), 8/10 patients showed a significant change in the front wave of the shear wave velocity, demonstrated by the intersection of the upper and lower slope trace (Fig. 5, Table 4). The intersection point corresponded to the transition zone between scar tissue and not-scarred myocardium.

All three patients having transmural scar showed abnormalities in both the inner and outer myocardial layers. Of the six patients with only endocardial scar, five showed localized inner myocardial layer abnormalities with shear-wave imaging and the single patient without scar also had normal shear-wave imaging (Table 2).

### Variability

The kappa coefficient of agreement between the two readers for the ten HCM subjects was 1.

## Discussion

The main finding of this study is that shear wave imaging can detect local variations in shear wave velocity presumably related to a variation in local myocardial properties (scar induced by septal reduction interventions).

This pilot study is based on the hypothesis that cardiac shear wave imaging can provide information about the local stiffness in the myocardial segments traversed by the shear waves. This type of semi-quantitative mapping of the myocardium could be used to generate myocardial elastograms, similar to the ones already used in other organs.

The relationship between shear wave velocity and tissue stiffness is from a physical point of view monotonic [1]. Naturally-occurring shear waves in the heart are produced during periods of variation in muscle tension (contraction or relaxation) and intracavitary pressure [3, 5, 11–14, 16], meaning that the shear wave velocity represents a mix of diastolic and contractile properties of the wall. Similar to the aortic valve closure, also the mitral valve closure generates a wave pattern that might appear in the TDI map. However, in previous studies [35, 36], the feasibility of the mitral shear wave detection was significantly lower than the aortic. Furthermore, a large number (8/10) of surgical reduction patients also received mitral valve reconstruction. The consequences of mitral repair on the shear waves induced by valve closure are yet unknown. Shear waves after mitral valve closure were therefore not considered in this study.

In both HCM groups, when measured in non-ablated myocardial segments the shear wave velocity was similar (5.3 ± 1.0 and 5.3 ± 0.7 m/s respectively, *p* = 0.9), and significantly higher than in healthy controls (3.6 ± 0.4 m/s, *p* < 0.0001). We have already reported parasternal aortic shear wave velocity values over 5 m/s in HCM patients, using the same high frame rate TDI methodology as in the current study [14]. Villemain et al., using externally induced shear waves during the diastolic phase, measured an average velocity of 3.5 m/s for adult HCM patients [10]. The difference in velocity with Villemain et al. probably relates to the role of myocardial contractility and parietal tension during the isovolumetric periods as compared to the diastasis. However, during the very short periods of time when shear waves are detected in the basal septum, this dynamic component is less important, meaning that a change in the propagation velocity should represent a variation in local tissue properties.

Given the method of detecting these waves [13, 14, 16] (cf. Figure 3), a variation in velocity is visible by a variation in the slope of the shear wave in the M-mode panel. A slope increase present in only one myocardial layer was interpreted as local (inner or outer layer) increase in tissue stiffness (tissue stiffening secondary to septal reduction therapy). Simultaneous presence of a velocity increase in the inner and outer layers was interpreted as transmural scar. Clinical color TDI is a duplex mode, the underlying 2D image confirmed that the variation in slope corresponded to the transition zone between scarred and not scarred myocardium.

Using as a study group HCM patients having undergone septal reduction allowed us not only to test the hypothesis that in the same subject we could detect normal versus abnormal shear wave propagation (as would have been the case in a simple model of myocardial infarction), but also that a difference could be detected between various degrees of pathological stiffness (non-ablated HCM myocardium and myocardial scar tissue in ablated segments). We used this subpopulation as a model to demonstrate that SWI is able to show local tissue stiffness variations, without investigating its added clinical value for this specific group of patients, as that was not our aim with this clinical pilot.

There were significant differences in age, body mass index and cardiac morphology between the normal volunteers and the patient groups. We chose to accept these differences because in practical terms it would be difficult to find healthy controls of similarly high age and BMI to our HCM groups who would still qualify as” normals” as per our inclusion/ exclusion criteria. Therefore, we preferred to compare to truly normal young subjects. As expected, based on our previous HCM study [14], HCM patients had higher shear wave velocities, as compared to normal individuals. The purpose of the healthy controls was to exclude that the velocity (slope) variation demonstrated in the septal reduction HCM group would be due to other factors than a variation in stiffness, and therefore be present in normal individuals.

The two patient groups had similar general features, except for gender distribution and ejection fraction, which was statistically lower in the septal reduction group (53 ± 14% versus 65 ± 9%, *p* = 0.03, Table 1), as expected in the presence of a septal motion abnormality. This was due to the selection criteria (stable age-matched HCM patients). The presence of layer-specific abnormalities only in group 3 (investigation group) implies that the variation in shear wave propagation is indicative of a local variation in tissue stiffness by scar, rather than any other phenomenon that would be typical for HCM such as geometry, thick septal wall or diffusely fibrotic tissue, as these would be equally present in groups 2 and 3.

We noticed a clear velocity variation in the inner layer in 8/10 HCM patients after septal reduction therapy, having a steeper slope in the part of the septum where tissue abnormalities were detected by standard imaging. This is in line with the expectation that these segments would be stiffer than the rest of the myocardium. The velocity variation was not visible in the HCM patients who have not had such an intervention, and it was also not visible in normal healthy volunteers, thus showing a good specificity of the technique. Three patients had a velocity change in both layers, interpreted as transmural scar. In these 3 patients, 3D echocardiography confirmed the presence of a transmural basal septal scar, demonstrating the accuracy of our diagnostic algorithm in this limited group of patients.

Theoretically, the spatial resolution of the clinical color TDI system should be sufficient to resolve different propagation velocities in a thick muscular layer [31]. The speckle size in color TDI in the axial direction was 1-3 mm and in the lateral 3-5 mm [13]. The smallest septal width in the parasternal long axis view was 8 ± 1 mm in normal volunteers, but 17 ± 4 m and respectively 19 ± 5 mm in HCM patients. In a muscular layer that is thicker than 15 mm, an axial resolution of 3 mm is sufficient to resolve two different adjacent regions. This had to be proven in a controlled setting by an in vitro study. By using a clinical shear wave elastography system (scanner 2), we computed the stiffness of the two adjacent layers in a tissue phantom resembling the in vivo scarred interventricular septum and calculated the corresponding shear wave velocities. Testing the shear wave quantification with the clinical system, we showed a shear wave propagation difference between the two different layers. The resulting velocities were similar to the expected computed values, thus confirming that our technique is able to distinguish and characterize different layers having velocities that are different, in the range of 4.5–6.3 m/s.

At 500 Hz and 3 cm length of the M-Mode line, one time frame (2 ms) corresponds to a maximal detectable velocity of 7.5 m/s. This also means that for a tissue with underlying shear wave velocity of 5 m/s, the system can detect an increase in velocity of around 2.5 m/s. This *velocity* resolution could be improved when using higher frame rates [13], yet the clinical scanner we used is not able to go beyond this –already relatively high- frame rate. We already know that average differences in shear wave velocities between normal myocardium and HCM are around 1.5–2 m/ s[14]. We expect that old fibrous scars have largely higher shear wave velocities, but these cannot be quantified with our current frame rate. At 500 Hz they present as vertical slopes, translating into velocities over 7.5 m/s. For this reason, we refrained from attempting to assess the two different velocities along the upper and lower direction of the front wave in the M-mode panel, when the two front wave lines were non-parallel. The velocity difference was detected by the angle shift, as described in Methods. The resolution of the angle shift method is 1.5–2 m/s, thus sufficient to detect expectedly large velocity differences.

### Limitations

This prospective proof-of-concept study enrolled only a limited number of patients, so we cannot draw statistically-founded conclusions. To further show the accuracy of the technique, results should be tested on a larger population.

High frame rate color TDI represents a tradeoff between spatial and temporal resolution and frame rate, as demonstrated above. The time resolution used in the current study (< 2 ms) may prove insufficient for very precise quantification of shear wave velocity variation in patients with very stiff ventricles (very high expected shear wave velocity).

Our semi-quantitative approach resulted in a very good interobserver agreement, but for clinical application, the assessment should be operator-independent. New research should focus on a robust method of automated velocity tracking from the DICOM frames, ideally at a higher frame rate.

Tissue remodeling or scars resulting from the different septal reduction techniques are not similar. Previous MRI studies demonstrated that by using the modern myectomy techniques myocardial scarring may be discrete and limited to a thin endocardial fibrous layer (as also found in our subjects, see Fig. 1) [30], that could be smaller than the resolution of some of the imaging methods compared here (3D echocardiography and SWI). Functional imaging by deformation (longitudinal strain) is more sensitive to the local deformation abnormality induced by scarring, giving diagnostic information in these cases.

### Potential clinical applications

Scar detection per se is not the purpose of this method, since it is better done by other imaging modalities. Being able to detect localized variations in tissue stiffness inside the myocardium however would potentially allow a quantitative mapping of the myocardial stiffness [37] Myocardial ischemia that starts in the inner layer [38], local inflammatory [18] or tumoral infiltrates [19], local deposition of abnormal proteins (focal amyloidosis) would all initially present as normal B-mode images in the echo, despite local stiffness variations, and might thus be missed and left untreated. Myocardial stiffness mapping, that could for example be rendered in color-coded stiffness maps, in a similar manner to that presently used in parenchymatous organs [1], would potentially allow a highly accessible and harmless method for early diagnosis in some of the conditions mentioned above.

We do not anticipate this goal to be achievable with the current clinical TDI application used in this work. Its ease of use makes it readily available for research, allowing to demonstrate that shear wave imaging is possible and clinically relevant. With advancement in technology even higher frame rates become available [11, 12]. In addition, changes in data processing will allow an increase in the velocity range that can be accurately measured.

## Conclusion

Local variations in stiffness, with myocardial remodeling post septal reduction therapy as model, can be detected by a local variation in the propagation velocity of naturally occurring shear waves.

## Supplementary information

**Additional file 1: Movie 1.** Multi-slice view of the left ventricle of the patient in Fig. 2, extracted from one full volume 3D acquisition. Note that in the classical apical views (upper panels) the inner layer scar is not visualized. The basal septum is investigated by using multiple transversal sections (lateral panel indicates the level of the slicing) and clearly demonstrated an inner layer scar (arrow central panel).

**Additional file 2: Movie 2.** Global longitudinal strain in apical 4 chambers view in one normal study subject. The acquisition is synchronized to the phonocardiography (upper curve) and ECG (lower curve).

## Data Availability

The datasets used and/or analyzed during the current study are available from the corresponding author on reasonable request.

## Abbreviations

ANOVA: Analysis of variance
ARF: Acoustic radiation force
BMI: Body mass index
DICOM: Digital imaging and communications in medicine
ECG: Electrocardiography
GLS: Global longitudinal strain
HCM: Hypertrophic cardiomyopathy
LSS: Longitudinal segmental strain
LV: Left ventricle
MRI: Magnetic resonance imaging
PCG: Phonocardiography
PLAX: Prasternal long axis view
SD: Standard deviation
SWI: Shear wave imaging
TDI: Tissue doppler imaging
